# Autonomic Modulation During Baseline and Recovery Sleep in Adult Sleepwalkers

**DOI:** 10.3389/fneur.2021.680596

**Published:** 2021-06-24

**Authors:** Geneviève Scavone, Andrée-Ann Baril, Jacques Montplaisir, Julie Carrier, Alex Desautels, Antonio Zadra

**Affiliations:** ^1^Center for Advanced Research in Sleep Medicine, Hôpital du Sacré-Cœur Montréal, Centre intégré universitaire de santé et de services sociaux du Nord-de-l'Île-de-Montréal (CIUSSS-NIM), Montreal, QC, Canada; ^2^Department of Psychology, Université de Montréal, Montréal, QC, Canada; ^3^The Framingham Heart Study, Boston University School of Medicine, Boston, MA, United States; ^4^Deparment of Psychiatry, Université de Montréal, Montréal, QC, Canada; ^5^Department of Neurosciences, Université de Montréal, Montréal, QC, Canada

**Keywords:** sleepwalking, somnambulism, sleep deprivation, heart rate variability, autonomic nervous system, slow-wave sleep, high frequency, parasomnia

## Abstract

Sleepwalking has been conceptualized as deregulation between slow-wave sleep and arousal, with its occurrence in predisposed patients increasing following sleep deprivation. Recent evidence showed autonomic changes before arousals and somnambulistic episodes, suggesting that autonomic dysfunctions may contribute to the pathophysiology of sleepwalking. We investigated cardiac autonomic modulation during slow-wave sleep in sleepwalkers and controls during normal and recovery sleep following sleep deprivation. Fourteen adult sleepwalkers (5M; 28.1 ± 5.8 years) and 14 sex- and age-matched normal controls were evaluated by video-polysomnography for one baseline night and during recovery sleep following 25 h of sleep deprivation. Autonomic modulation was investigated with heart rate variability during participants' slow-wave sleep in their first and second sleep cycles. 5-min electrocardiographic segments from slow-wave sleep were analyzed to investigate low-frequency (LF) and high-frequency (HF) components of heart rate spectral decomposition. Group (sleepwalkers, controls) X condition (baseline, recovery) ANOVAs were performed to compare LF and HF in absolute and normalized units (nLF and nHF), and LF/HF ratio. When compared to controls, sleepwalkers' recovery slow-wave sleep showed lower LF/HF ratio and higher nHF during the first sleep cycle. In fact, compared to baseline recordings, sleepwalkers, but not controls, showed a significant decrease in nLF and LF/HF ratio as well as increased nHF during recovery slow-wave sleep during the first cycle. Although non-significant, similar findings with medium effect sizes were observed for absolute values (LF, HF). Patterns of autonomic modulation during sleepwalkers' recovery slow-wave sleep suggest parasympathetic dominance as compared to baseline sleep values and to controls. This parasympathetic predominance may be a marker of abnormal neural mechanisms underlying, or interfere with, the arousal processes and contribute to the pathophysiology of sleepwalking.

## Introduction

Sleepwalking, or somnambulism, is a non-rapid-eye movement (NREM) sleep parasomnia characterized by behavioral episodes of varying complexity, usually initiated during slow-wave sleep during the first third of the night (1, 2). Sleepwalking affects up to 4% of adults (3, 4) and represents a leading cause of sleep-related violence and self-injury (5–7). Moreover, sleepwalking in adults has been found to be associated with daytime impairments, including reduced well-being and excessive somnolence (1, 8, 9).

Because sleep deprivation results in a rebound of slow-wave sleep during recovery sleep, it increases the frequency and complexity of sleepwalking episodes in predisposed patients, while having no such effect in normal controls (1, 10). Sleepwalkers have also been shown to suffer from inability to maintain stable, consolidated NREM sleep, especially during the first sleep cycle, and this inability likely reflects an abnormality in the neural mechanisms responsible for regulating slow-wave sleep (1, 9). For these reasons, sleepwalking is hypothesized to be a disorder of arousal as well as one of slow-wave sleep regulation (1, 9). However, the exact nature of the mechanisms that give rise to sleepwalking remains unclear.

The neural systems regulating sleep are anatomically and physiologically closely interrelated with the autonomic nervous system (11, 12). The autonomic nervous system regulates the body homeostasis, including blood pressure, breathing, temperature, digestion, etc., via its two branches, the sympathetic and parasympathetic nervous systems, each having opposing effects. NREM sleep, and especially slow-wave sleep, is characterized by a strong parasympathetic autonomic modulation concomitant with decreased sympathetic modulation as compared to wakefulness (11, 13–16). Moreover, there is some evidence to suggest that sleep deprivation affects the interplay between sleep depth and autonomic function in healthy controls (17). Therefore, investigating autonomic nervous system functioning in sleepwalkers following a period of sleep deprivation may provide insights into the neural pathophysiological mechanisms involved in sleepwalkers' slow-wave sleep regulation. A few studies having measured heart rate in sleepwalkers found autonomic changes suggestive of an abrupt sympathetic activation before episodes, including during slow-wave sleep (18–20). These preliminary reports suggest that autonomic dysfunctions might play a role in pathophysiology of sleepwalking. However, the possible significance of autonomic regulation in sleepwalkers' slow-wave sleep and its response to sleep deprivation have yet to be investigated.

Because the sympathetic and parasympathetic systems have opposing effects on heart rate, measuring heart rate variability (HRV) allows to estimate autonomic influences on cardiac function. The spectral frequency decomposition of the HRV signal provides key information contained in low-frequency (LF) and high-frequency (HF) bands. Whereas, it is widely accepted that the HF band represents respiratory sinus arrhythmia, and thus, is an index of parasympathetic modulation, the autonomic contribution to the LF band is more complex (11, 21). Although the LF band has been traditionally regarded as a marker of sympathetic activity, it is now known that parasympathetic modulation contributes partially to this band as well (22, 23). In fact, the LF band has been described as a marker of central modulation of the autonomic nervous system (rather than effective tone) and baroreflex functioning (21). While the LF/HF ratio has been widely used to measure the sympathovagal balance, interpretations of this ratio remain controversial because of the limitations of the LF band and because both branches of the autonomic nervous system can be activated simultaneously (11, 21, 22). Despite questions regarding its physiological interpretation, the LF/HF ratio remains a valuable index to better understand sleep autonomic control (11).

To our knowledge, only one study investigated frequency-domain HRV variables in sleepwalkers (*n* = 10) in comparison to controls. While the authors did not find between-group differences during sleep as a whole, they did not investigate specific sleep stages (19). Given the role of slow-wave sleep in sleepwalking pathophysiology (1, 9), measuring the LF and HF bands, and the LF/HF ratio specifically during sleepwalkers' slow-wave sleep, both before and after a period of sleep deprivation, could provide valuable insights into autonomic modulation in this clinical population.

Since a sympathetic activation during sleepwalkers' slow-wave sleep could be associated with their atypical arousal process and translated into changes in frequency-domain HRV variables (11, 24), we investigated the autonomic nervous system modulation during sleep in sleepwalkers and controls. Specifically, this modulation was studied during normal and recovery slow-wave sleep following 25 h of sleep deprivation, with a focus on the first sleep cycle when most behavioral episodes occur. We hypothesized that when compared to controls, sleepwalkers would show HRV patterns suggestive of a balance shifting toward sympathetic predominance (increased LF and LF/HF ratio; decreased HF) during slow-wave sleep, especially during their post-sleep deprivation recovery sleep.

## Materials and Methods

Fourteen adult sleepwalkers (nine women, five men, mean age: 28.1 ± 5.8 years) and 14 sex- and age-matched controls (nine women, five men, 27.8 ± 6.0 years) were investigated. Sleepwalkers included were referred to our sleep disorders clinic by their treating physician. They had a history of chronic and frequent sleepwalking (>3 years, >1 episode/month) and met *International Classification of Sleep Disorders-III* diagnostic criteria for sleepwalking (2). All participants underwent a baseline night of polysomnography (PSG) directly followed by a 25 h sleep deprivation. Afterward, recovery sleep, also recorded with PSG, began in the morning. Some participants of this cohort were also included in previous studies by our group evaluating the impact of sleep deprivation on sleepwalkers' sleep and episodes (10, 25, 26).

Exclusion criteria for all participants consisted of 1) the presence of another sleep disorder, an apnea-hypopnea index >10 events/h, or periodic leg movements during sleep >15 events/h; 2) the presence of any major psychiatric disorder; 3) the presence or history of a neurologic disorder, including traumatic brain injury and epilepsy; 4) the use of medications that could influence the electroencephalogram (EEG), sleep architecture, autonomic function, motor activity during sleep, or daytime vigilance; 5) a transmeridian traveling or shift work in the 3 months preceding the study; 6) a body mass index >30 kg/m^2^; and 7) sleep efficiency <80% or a sleep latency >30 min during baseline PSG.

All 28 participants had a minimum of two complete sleep cycles during both their normal baseline sleep and their recovery sleep, and had adequate electrocardiogram (EKG) signals to allow R-wave detection of the QRS complex to measure heartbeats in at least one 5 min segment of continuous slow-wave sleep without signal interruption (e.g., EEG arousal, leg movements, respiratory events) in the first two sleep cycles. The protocol was approved by the hospital's Ethics Committee and all participants provided written consent prior to the study.

### PSG Recordings

In the baseline experimental night, lights off was between 10:00 p.m. and 12:00 a.m. (midnight) and wake time between 6:00 a.m. and 8:00 a.m., depending on each participant's habitual sleep-wake cycle. After the baseline recording, sleepwalkers were instructed to go about their regular daytime activities but were forbidden from taking naps. They returned to the laboratory in the evening for the sleep deprivation protocol and remained under constant supervision for the night. Control subjects were submitted to the same protocol but remained in the laboratory under a constant routine condition for the diurnal period, as they were healthy controls for another sleep study as well. Recovery sleep was scheduled the following morning for all participants, 1 h after their previous wake time (i.e., following 25 h of wakefulness). Participants were prohibited from consuming alcohol, caffeine, or other stimulating substances the day prior to and during all laboratory procedures. Further details on this sleep deprivation protocol have been published elsewhere (10).

PSG recordings were conducted on a 32-channel Grass polygraph (sensitivity at 7 μV, bandpass at 0.3–100 Hz; Grass Instruments, Quincy, MA). Signals were relayed and digitized at a sampling rate of 256 Hz, and digitally filtered with an upper cut-off frequency of 100 Hz using a commercial software (Harmonie, Stellate Systems, Montreal, Canada). EEG recordings and electrodes placement were performed according to the international 10–20 system with a linked-ear reference and included electro-oculograms, submental electromyography, surface electromyography of the bilateral anterior tibialis, and continuous recording of 3-lead EKG (standard DI, DII and DIII). Respiration was also monitored using an oronasal canula and a thoracoabdominal plethysmograph, whereas oxygen saturation was recorded with a finger pulse oximeter. Sleep stages were scored according to standard criteria (27). A sleep cycle was defined as a NREM sleep period lasting at least 15 min followed by an REM sleep period lasting at least 5 min, except for the first REM episode which could have been shorter. Sleep time over the first two sleep cycles was calculated from sleep onset to the end of the second cycle. Sleep efficiency was the total sleep time over the first two cycles divided by the total sleep period from lights off to the end of the second sleep cycle. Slow-wave activity was averaged on C3 and C4 during the first two cycles and assessed as previously described (25).

### Spectral Components of Heart Rate Variability

Slow-wave sleep has been viewed as a stable and robust recording condition to assess HRV. In fact, the reduced inputs from external stimuli, limited bodily movements, being in a supine position, and stable and regular respiratory cycles that characterize slow-wave sleep are viewed as representing a unique opportunity to measure HRV indexes with few confounding factors (14).

The EKG was analyzed in a two-step process to obtain frequency-domain indexes of the HRV during slow-wave sleep, or N3 sleep stage. 5 min EKG segments with stationary signals were first selected blind by two of the authors (G.S. and A.A.B.) from slow-wave sleep during both baseline and recovery sleep. The 5 min length for selected segments was chosen as this duration was suggested to be the ideal time window to measure HRV during sleep to avoid artifactual or interruptive events and to facilitate standardization across studies (11). Selected segments were free of EEG arousals, movement artifacts such as leg movements during sleep, and respiratory events during and 40 s before and after each segment. Segments were still selected if a maximum of two N2 20-s epochs were present. However, no segments were selected containing epochs of REM sleep, and no segments were selected 5 min away from any REM sleep epoch. All appropriate 5 min segments corresponding to these criteria were selected from the first two sleep cycles. If more than one segment met the criteria within the same cycle, all were selected provided the segments were separated by 15 min or more in order to obtain a wider sampling of slow-wave sleep. Whenever more than one EKG segment was selected from the same sleep cycle, values for cardiac variables were averaged into a single score for that cycle. Each participant thus had a single score for each HRV variable for each cycle for both baseline and recovery sleep.

HRV was investigated using CardioLab (Fondazione S. Maugeri, Italy). Detection of R-wave peaks representing each heartbeat was first automatically detected and instances of arrhythmia or signal artifacts subsequently removed. Results from the software's automatic signal detection were then verified and confirmed by visual inspection. Frequency-domain components of the HRV assessed were absolute LF and HF bands, the LF/HF ratio, and normalized LF and HF bands (nLF, nHF) on total power. Frequency-domain components were quantified with an autoregressive decomposition algorithm to compute spectral peak powers, central frequencies, and classified into LF (0.04–0.15 Hz) and HF (0.15–0.4 Hz) bands. Because normalized measures are mathematically redundant and equal carriers of information with the LF/HF ratio when the denominator of their normalization is LF+HF, we opted for normalizing the bands on total power (from very low frequency and very high frequency) (28). The normalization was made to assess the relative contribution of the LF and HF bands to the total power and to remove the very large inter-subject variability that typically characterizes measures of spectral HRV (28).

Of note, although frequency-domain variables should be interpreted with caution as described above, for the purpose of this study, a shift in the LF/HF ratio or normalized bands will be referred to as a “predominance.”

### Statistical Analyses

Statistics were performed with SPSS version 25 (IBM, Armonk, NY). Group (sleepwalkers, controls) ^*^ Condition (baseline, recovery) ANOVAs were performed for sleep architecture and all HRV variables during slow-wave sleep in both the first cycle as well as the second sleep cycle, as a secondary goal was to evaluate whether HRV changes extend to the night's second period of slow-wave sleep. HRV variables in statistical models were transformed using natural logarithm to normalize the distribution as is commonly done for these metrics (23). Group ^*^ Condition ANOVAs were also performed for sleep architecture characteristics in the first and second sleep cycles grouped together. Significant interactions were decomposed using *post-hoc* comparisons. *P-*values <0.05 were considered significant. A false-discovery rate (FDR) correction was applied to Group ^*^ Condition ANOVAs for interactions with HRV variables.

A series of *post-hoc* analyses were performed to better characterize the association between HRV variables, markers of disrupted slow-wave sleep and elevated arousals, and sleepwalking episodes. First, correlations between interactions terms (Group ^*^ HRV variables during recovery slow-wave sleep of cycle 1) and sleep architecture and episodes were conducted to evaluate if groups differed in their associations with HRV and sleep characteristics. Second, we correlated interaction terms (slow-wave activity ^*^ HRV variables during recovery slow-wave sleep of cycle 1) with sleep architecture and episodes in sleepwalkers to assess whether HRV variables and slow-wave activity showed interactions in their association with sleep parameters associated with sleepwalking. Finally, we re-ran Group ^*^ Condition interactions while adjusting for slow-wave activity.

## Results

### Sleep Architecture of Sleepwalkers

During baseline PSG recordings, 67% of sleepwalkers experienced a somnambulistic episode, with 50% of sleepwalkers having more than one. During recovery sleep, all sleepwalkers experienced a somnambulistic episode, with 75% of sleepwalkers having more than one. As expected, no somnambulistic episodes were observed in the control group during baseline or recovery sleep.

Sleepwalkers and controls differed on a few sleep variables assessed during the first two sleep cycles. Table 1 presents the means and standard deviations of sleep variables as well as summaries from the Group ^*^ Condition ANOVAs. A Group ^*^ Condition interaction was observed for slow-wave activity (*F* = 4.9, *p* < 0.05). *Post-hoc* comparisons revealed that sleepwalkers had significantly lower slow-wave activity than did the controls both at baseline and during recovery sleep (*p* < 0.05). In fact, only controls showed a significant increase in slow-wave activity from baseline to recovery sleep (*p* < 0.001). We also observed a Group ^*^ Condition interaction for the percentage of REM sleep (*F* = 4.3, *p* < 0.05), but *post-hoc* comparisons did not reveal significant effects.

**Table 1:** Sleep characteristics of sleepwalkers and control subjects from sleep onset to the end of the second sleep cycle.

**Sleep measures**	**Sleepwalkers (*****n*** **= 14)**		**Controls (*****n*** **= 14)**		***P* < 0.05**	***Post-hocs***
	**Baseline [1]**	**Recovery [2]**	**Baseline [3]**	**Recovery [4]**		
Sleep latency, min	12 (6.7)	2.2 (2.5)	9.3 (5.5)	2.6 (2.0)	b	
Sleep time, min	245.2 (54.0)	196.0 (43.1)	221.4 (39.5)	197.4 (38.4)	b	
Sleep efficiency, min	91.9 (5.6)	93.2 (5.5)	96.5 (3.0)	97.4 (1.9)	a	
N2 (min)	125.1 (33.5)	82.9 (23.5)	95.4 (32.9)	73.4 (25.5)	a; b	
N2 (%)	50.9 (6.8)	42.2 (6.4)	42.4 (9.6)	37.4 (11.0)	a; b	
N3 (min)	58.7 (23.1)	63.2 (18.8)	65.9 (18.7)	77.7 (25.1)		
N3 (%)	24.4 (9.7)	32.7 (8.4)	30.1 (7.9)	39.2 (10.3)	a; b	
REM (min)	41.8 (12.7)	39.5 (19.4)	47.8 (17.0)	39.3 (17.7)		
REM (%)	17.1 (4.1)	19.7 (8.0)	21.9 (7.9)	19.8 (7.3)	c	ns
Slow-wave activity	1192.7 (419.3)	1249.7 (452.4)	1665.0 (605.1)	1857.9 (702.7)	a; b; c	1 <3,4; 2,3 <4
Arousal index	4.3 (1.8)	3.2 (1.9)	3.4 (1.9)	2.5 (0.9)	b	
Arousal index in N3	3.4 (3.2)	3.5 (2.8)	1.8 (2.1)	1.1 (0.9)	a	

*Results are presented as mean (standard deviation)*.

a *Significant group effect;*

b *Significant condition effect;*

c *Significant interaction*.

Main group effects were also observed. Compared to controls' values at baseline and during recovery sleep, sleepwalkers showed lower sleep efficiency (*F* = 11.6, *p* < 0.01), lower percentage of N3 sleep (*F* = 4.7, *p* < 0.05), more arousals during N3 sleep (*F* = 6.4, *p* < 0.05), and a higher percentage and duration of N2 sleep (*F* = 6.4, *F* = 6.1; *p* < 0.05).

Regarding main condition effects, when compared to sleepwalkers' and controls' baseline, recovery sleep revealed a significantly shorter sleep latency (*F* = 50.2, *p* < 0.001), shorter sleep time during the first two sleep cycles (*F* = 10.5, *p* < 0.01), lower percentage and duration of N2 sleep (*F* = 12.4, *F* = 17.6; *p* < 0.01), higher percentage of N3 sleep (*F* = 19.2, *p* < 0.001), and a lower arousal index (*F* = 7.0, *p* < 0.01).

Further details on the sleep characteristics and behavioral episodes in this patient cohort have been published elsewhere (10, 25, 26).

### Frequency-Domain HRV Markers

**Figure 1:**
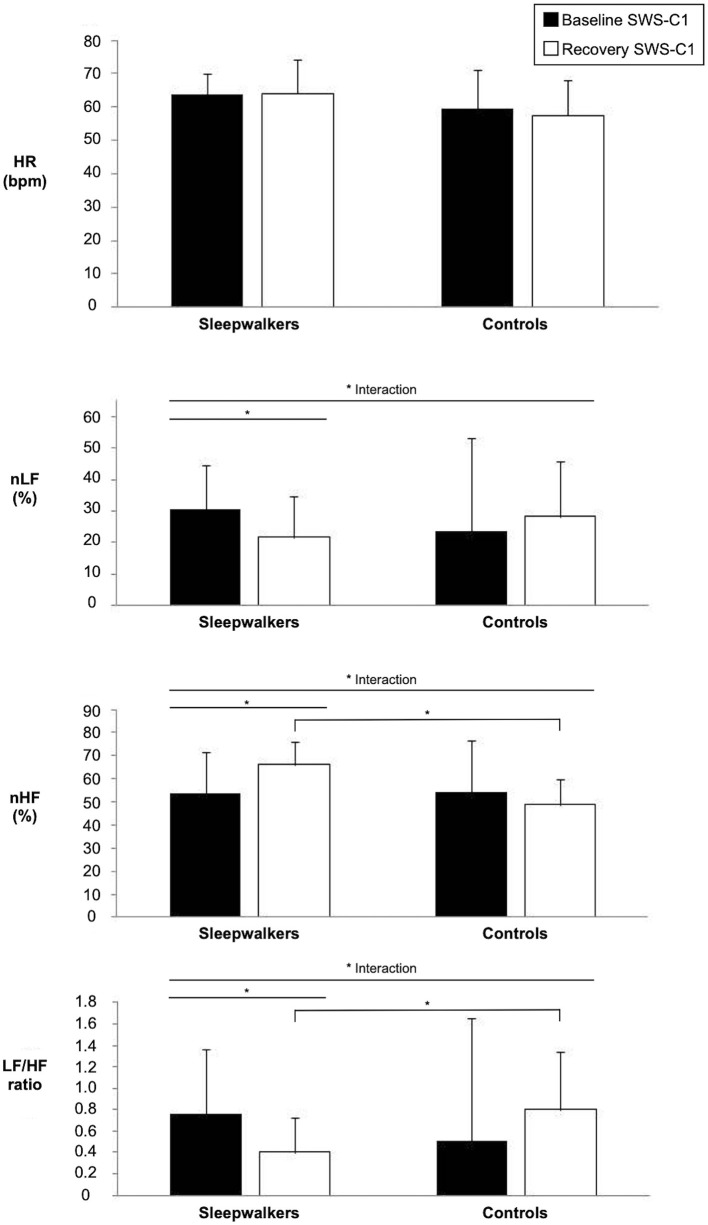
Significant interactions of HRV variables during slow-wave sleep in the first sleep cycle of sleepwalkers and controls for baseline and recovery sleep. Bars represents means and standard deviations. (*) show significant findings. HRV, heart rate variability; HR, heart rate; nLF, low frequency in normalized units as a percentage of total power; nHF, high frequency in normalized units as a percentage of total power SWS-C1, slow-wave sleep first cycle.

**Figure 2:**
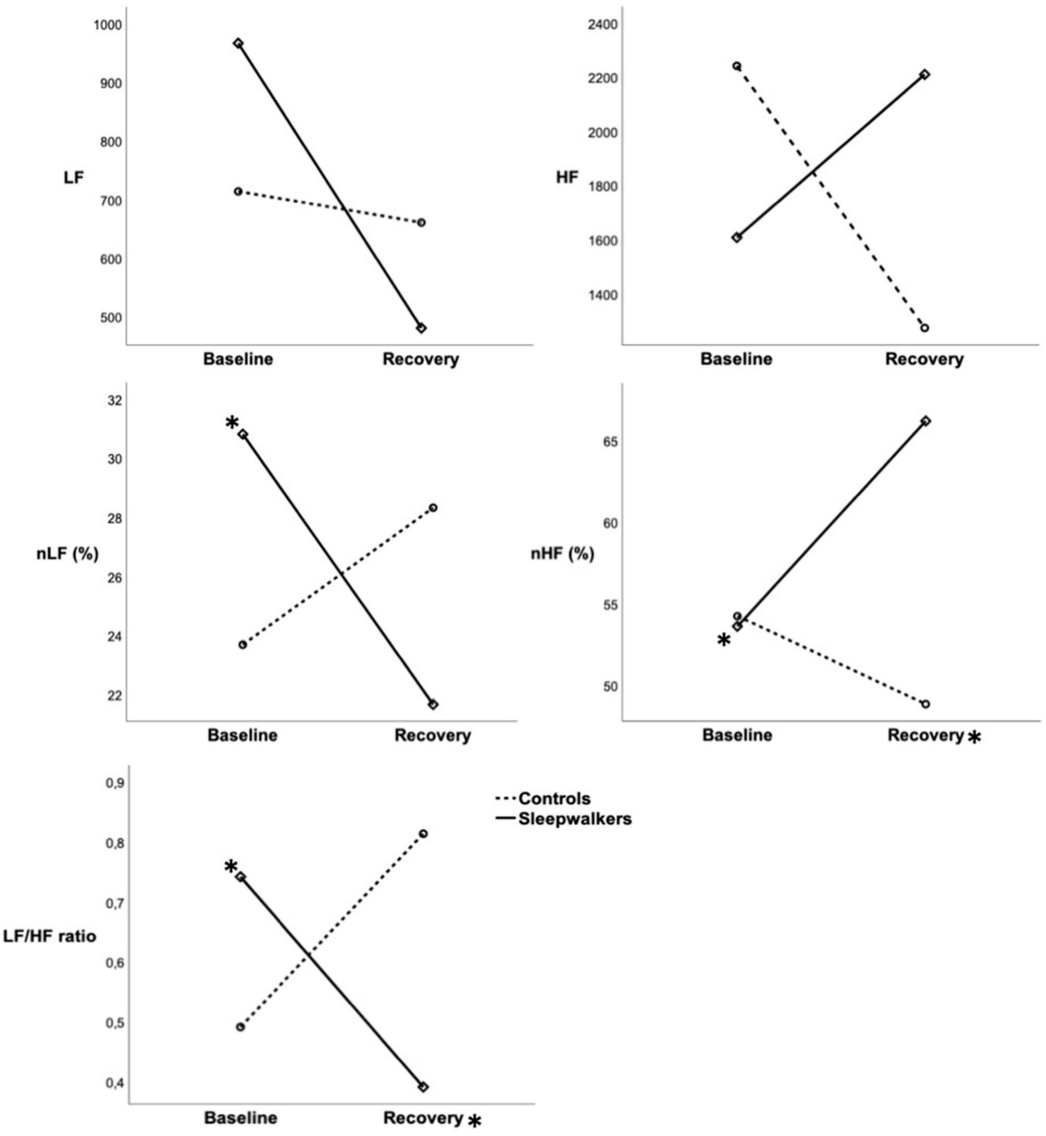
Mean HRV variables during slow-wave sleep in the first sleep cycle of sleepwalkers and controls during baseline and recovery sleep. Full lines represent sleepwalkers whereas dashed lines represent controls. (*) next to a line indicate a significant difference between baseline and recovery sleep in a given group whereas (*) next to a condition indicate a significant difference between sleepwalkers and controls. HRV, heart rate variability; HR, heart rate; LF, low frequency; nLF, low frequency in normalized units as a percentage of total power; HF, high frequency; nHF, high frequency in normalized units as a percentage of total power SWS-C1, slow-wave sleep first cycle.

Significant Group ^*^ Condition interactions were observed during the first sleep cycle for nLF and nHF as well as for the LF/HF ratio (Table 2). *Post-hoc* comparisons revealed that during the first cycle of recovery slow-wave sleep, sleepwalkers showed a lower LF/HF ratio and higher nHF compared to controls, although no group difference was observed at baseline. When compared to their slow-wave sleep at baseline, sleepwalkers' recovery slow-wave sleep presented lower nLF and LF/HF ratio as well as higher nHF during the first cycle. These significant interactions and *post-hoc* tests are presented in Figures 1, 2. No significant effect was found between the controls' baseline and their recovery slow-wave sleep.

**Table 2:** Frequency HRV variables in sleepwalkers and control subjects during the first two cycles of slow-wave sleep for baseline and recovery sleep.

**HRV**	**ANOVA: F(df1, df2)**, ***P*****-value**			**Sleepwalkers (*****n*** **= 14)**		**Controls (*****n*** **= 14)**		***Post-hocs***	**Effect sizes (d)**	
	Interaction^*^	**Group**	**Condition**	**Baseline [1]**	**Recovery [2]**	**Baseline [3]**	**Recovery [4]**		**[1] vs. [2]**	**[2] vs. [4]**
**Slow-wave sleep during cycle 1**										
LF	2.57(1,26), 0.287	0.31(1,26), 0.583	0.11(1,26), 0.748	967.25 (1733.02)	481.61 (401.00)	715.11 (928.90)	661.70 (492.58)		0.386	0.401
nLF	9.49(1,26), **0.025**	0.03(1,26), 0.868	0.92(1,26), 0.338	30.83 (13.63)	21.69 (13.07)	23.71 (12.30)	28.34 (11.90)	1>2	0.684	0.532
HF	1.98(1,26), 0.287	0.01(1,26), 0.923	0.38(1,26), 0.544	1609.79 (2353.98)	2212.02 (3058.07)	2243.58 (3459.36)	1275.19 (1104.47)		0.221	0.407
nHF	7.85(1,26), **0.030**	2.26(1,26), 0.145	0.47(1,26), 0.500	53.65 (17.69)	66.21 (14.05)	54.29 (12.83)	48.90 (15.43)	2>1,3,4	0.786	1.173
LF/HF	12.82(1,26), **0.010**	0.77(1,26), 0.389	0.51(1,26), 0.481	0.74 (0.60)	0.39 (0.33)	0.49 (0.32)	0.81 (0.78)	2 <1,4	0.773	0.701
**Slow-wave sleep during cycle 2**										
LF	2.07(1,23), 0.287	0.82(1,23), 0.374	0.98(1,23), 0.332	717.52 (540.91)	407.73 (325.35)	1901.76 (4987.75)	744.35 (787.70)		0.694	0.559
nLF	0.88(1,21), 0.450	0.51(1,21), 0.485	1.69(1,21), 0.208	33.95 (30.57)	28.55 (19.31)	31.99 (18.70)	31.95 (19.57)		0.211	0.175
HF	1.72(1,23), 0.290	0.001(1,23), 0.973	1.21(1,23), 0.283	1616.79 (1802.94)	1395.74 (1818.30)	3050.67 (5888.67)	1314.90 (1157.00)		0.122	0.053
nHF	0.38(1,21), 0.608	0.15(1,21), 0.719	0.44(1,21), 0.513	55.37 (23.07)	56.42 (23.04)	52.14 (17.77)	48.97 (19.68)		0.046	0.348
LF/HF	0.22(1,22), 0.643	0.25(1,22), 0.624	0.07(1,22), 0.794	0.96 (1.16)	1.23 (2.42)	0.88 (0.99)	1.16 (1.50)		0.142	0.035

*Although raw variables are presented in the table, all statistics were performed on HRV variables following natural logarithm transformations. Cohen's d values are presented for effect sizes, where a value around 0.2 is considered a small effect, a value close to 0.5 is considered a medium effect, and a value around 0.8 is considered a large effect*.

* *P-values for the interaction term are FDR-corrected. Significant post-hocs (p < 0.05) are presented for significant interaction terms. Bold values represent significant findings. HRV, heart rate variability; LF, low frequency band; nLF, normalized low frequency band; HF, high frequency band; nHF, normalized high frequency band*.

No main effects were observed for nLF, nHF or the LF/HF ratio (see Table 2). No significant interactions or main effects were observed for total power, absolute LF and absolute HF. Although interactions were non-significant for absolute measures during the first sleep cycle of slow-wave sleep, the direction of effects was consistent with the significant normalized measures, with medium effect sizes (Table 2, Figure 2). The absence of statistically significant differences in the absolute measures may be due, in part, to large inter-subject variability as evidenced by the large standard deviations in these measures.

During the first slow-wave recovery sleep cycle, group by HRV variables interactions were observed when correlating with N3 awakenings (*p* < 0.05), but not for N3 sleep stage duration, N3 micro-arousals or number of sleepwalking episodes. A lower LF/HF ratio during recovery slow-wave sleep of sleepwalkers correlated with an increase in the number of N3 awakenings from baseline to recovery sleep (delta between recovery and baseline, *r* = −0.58, *p* < 0.05), while this was not observed in controls. No significant associations were observed between HRV variables with the number of sleepwalking episodes during recovery slow-wave sleep. However, we could not investigate whether HRV variables correlated with the episode occurrence since all sleepwalkers experienced at least one episode during their recovery sleep. On the other hand, EEG slow-wave activity and nLF in sleepwalkers' first recovery slow-wave sleep cycle interacted together in their association with the increase of episodes from baseline to recovery sleep (*p* < 0.05): lower nLF was more strongly associated with the increased number of episodes from baseline to recovery in sleepwalkers that presented with lower levels of slow-wave activity.

When slow-wave activity was entered as a covariate in the Group ^*^ Conditions interactions, the interactions for the LF/HF ratio and nLF remained significant, whereas the interaction for nHF was no longer statistically significant.

None of the Group ^*^ Condition effects remained significant during the second sleep cycle. This finding suggests that changes in sleepwalkers' autonomic modulation occur mostly during their first cycle slow-wave sleep, similar to their other atypical sleep characteristics (increased arousals, diminished slow-wave activity) (1, 29).

Overall, the interactions suggest that sleepwalkers show an autonomic modulation that is parasympathetic-dominant during the first cycle recovery slow-wave sleep compared to controls as well as to their own baseline.

## Discussion

The aim of this study was to investigate HRV as a marker of autonomic modulation to gain insight into pathophysiologic mechanisms underlying sleepwalkers' atypical slow-wave sleep. Although we did not observe significant differences between sleepwalkers and controls during baseline slow-wave sleep, sleepwalkers displayed higher nHF and lower nLF and LF/HF ratio during recovery first cycle of slow-wave sleep compared to controls as well as to their own baseline sleep recordings. Contrary to our prediction, these findings suggest that sleep deprivation promotes a parasympathetic dominance of the autonomic modulation of the heart during sleepwalkers' slow-wave sleep. Because patients' somnambulistic episodes are more frequent and complex after extended sleep deprivation (10), our findings suggest that abnormal autonomic modulation may be involved in the pathophysiology of sleepwalking.

### Altered Autonomic Regulation During Recovery Slow-Wave Sleep in Sleepwalkers

As previously noted in other cohorts (30), sleepwalkers in the present study showed abnormal slow-wave sleep characterized by lower slow-wave activity and more arousals from slow-wave sleep when compared to controls. These findings support the view that sleepwalking is associated with a difficulty in maintaining consolidated slow-wave sleep (1). Our novel finding of lower LF/HF ratio suggesting an autonomic modulation that is parasympathetic predominant also points to an altered regulation of slow-wave sleep. The only previous study of autonomic function in 10 sleepwalkers vs. 10 controls found no group differences in frequency-domain metrics during participants' overall sleep (i.e., without examining specific sleep stages, nor sleep deprivation) or during wakefulness (19). These findings are consistent with those from the present study since we did not observe HRV change at baseline, suggesting that sleepwalkers may exhibit abnormal autonomic regulation as a function of homeostatic sleep pressure following extended sleep deprivation. In healthy controls, and as observed in the present study, sleep deprivation increases homeostatic sleep pressure, and thus, slow-wave sleep and slow-wave activity (17). This pattern was also previously reported in sleepwalkers after 38 h of sleep deprivation (31). However, using a 25 h sleep deprivation protocol, we did not observe a significant increase in slow-wave activity during sleepwalkers' recovery slow-wave sleep, which also suggests poorer consolidated slow-wave sleep in this population.

In the present study, the effect of a 25 h sleep deprivation, which results in daytime recovery sleep, differed between controls and sleepwalkers on HRV metrics. Whereas, sleep deprivation was associated with HRV metrics suggestive of a parasympathetic predominance in sleepwalkers' recovery slow-wave sleep, this association was not observed in controls. Although deeper slow-wave sleep is normally characterized by a predominance of parasympathetic modulation with elevated HF (13–16, 32), results from one study suggest that extended wakefulness is associated with elevated sympathetic activity during recovery sleep despite longer slow-wave sleep (17), which could explain our findings in controls. These varying results in healthy controls may stem from a mixture of effects from circadian, homeostatic and extended wakefulness factors. That said, while the effect of sleep deprivation on autonomic modulation during recovery sleep, and especially slow-wave sleep, remains unexplored in the literature, our current findings suggest that the effect differs between sleepwalkers and healthy controls.

Why sleepwalkers present different patterns of autonomic modulation during recovery slow-wave sleep than do controls remains unclear. Since structures of the central nervous system that regulate sleep and autonomic function are closely interrelated (11, 12), this difference in autonomic function in sleepwalkers might be the consequence of abnormal functioning of central neural regulatory systems. Moreover, given that a genetic predisposition likely plays an important role in the development of somnambulism (33–36), genetic factors could underlie the differential autonomic modulation observed in sleepwalkers after sleep deprivation. In fact, it was suggested that certain genes can affect the interaction between autonomic and slow-wave sleep regulation (17). It is possible that genetic contributions to sleepwalking include genes associated with autonomic functioning.

### Autonomic Modulation, Abnormal Slow-Wave Sleep and Occurrence of Episodes in Sleepwalkers

Whereas, sleepwalkers' overall slow-wave sleep showed HRV metrics suggestive of parasympathetic predominance in our study, sleepwalkers' slow-wave sleep prior to behavioral episodes or arousals was previously associated with heart rate markers suggesting sympathetic activation. In one study, increased HRV total spectral power was found to increase during the 5 min preceding episodes in 10 sleepwalkers in a normal sleep condition (19). Another study under normal sleep conditions showed that arousals during slow-wave sleep in 38 predisposed individuals were associated with an abrupt heart rate acceleration (18). A similar finding was found for motor arousals during slow-wave sleep associated with sleepwalking or sleep terrors in 20 participants, with arousals being preceded by an abrupt heart rate acceleration (20). Overall, our present findings suggest that this activation prior to episode occurrence does not appear to extend to entire periods of slow-wave sleep.

Taken together, these previous findings on heart rate acceleration as well as the findings suggestive of parasympathetic dominance observed in the present study during sleepwalkers' recovery slow-wave sleep point to altered autonomic modulation that may be a marker of the disorder's underlying pathophysiology, or could play a more direct role in the occurrence of somnambulistic episodes. Indeed, we observed that HRV variables suggestive of parasympathetic dominance were associated with increased slow-wave sleep awakenings and interacted with slow-wave activity in their association with increased frequency of episodes from baseline to recovery sleep. Although the directionality of the association between autonomic modulation and sleep-related arousals remains subject to debate, some findings show that normal sleep-related arousals and EEG changes are preceded by autonomic fluctuations (11), suggesting that autonomic modulation may play a role in the initiation of arousals. In fact, an interaction between parasympathetic predominance during slow-wave sleep and transitions from sleep to wakefulness characterized by sudden sympathetic activation may contribute to behavioral manifestations associated with sleepwalking. This pattern is similar to what is observed with slow-wave activity in sleepwalkers. Specifically, although sleepwalkers present with decreased slow-wave activity (30), as seen in the present study, somnambulistic episodes are often characterized by relatively abrupt increases in slow oscillations and slow delta frequency seen immediately prior to episodes' occurrence (25). The dissociation between parasympathetic dominance during slow-wave sleep and potentially abrupt sympathetic activation during arousals could signal the concomitant presence of features associated with both sleep and wakefulness during sleepwalkers' abnormal arousal reactions. Consistent with this line of reasoning, a study of EEG functional connectivity in sleepwalkers found that somnambulistic episodes are preceded by brain processes suggestive of the coexistence of arousal and sleep (37). The coexistence of arousal and sleep mechanisms in disorders of arousals were also suggested by studies using high density EEG source modeling, EEG current density imaging, single-photon emission computed tomography, and intracerebral EEG (38–41).

It remains unclear whether altered autonomic modulation during recovery slow-wave sleep is only a marker of abnormal neural circuitry in predisposed individuals or if it plays an active role in precipitating somnambulistic episodes. Some psychotropic medications, most notably zolpidem, are known to facilitate sleepwalking in individuals with no past history of the disorder (42). During sleep, zolpidem has been shown to promote the reduction of blood pressure (43), a normal phenomenon known as “dipping” that is viewed as being a consequence of lower sympathetic outflow during sleep (12, 44). Moreover, zolpidem reduces levels of epinephrine and norepinephrine (43), two neurotransmitters secreted following sympathetic activation. This suggests that a potential shift of the sympathovagal balance observed with zolpidem could inform sleepwalking pathophysiology. Similarly, sleepwalking can arise through the use of beta blockers (42), whose main mechanism of action is competitive beta adrenergic receptor antagonists and thus, block sympathetic effects. More recently, methylphenidate, a stimulant that increases the sympathovagal balance toward sympathetic modulation (45), has been used to treat two patients presenting with a history of chronic and severe sleepwalking (46). Taken together, these findings suggest that autonomic modulation, and especially parasympathetic dominance during slow-wave sleep, might play a role in the pathophysiology of sleepwalking. Whether or not therapeutic avenues known to affect autonomic modulation could also impact sleepwalking remains to be investigated.

### Limitations

Sleepwalkers were allowed to leave the laboratory setting for the day after their baseline sleep assessment whereas controls remained at the laboratory in constant routine. Thus, sleepwalkers may have experienced greater daytime activity levels than controls before returning to the lab for the sleep deprivation protocol. However, greater levels of daytime activity would have been expected to be associated with increased slow-wave activity (47, 48), which was not observed in sleepwalkers, suggesting that our findings are not accounted for by different daytime activity levels. Because recovery sleep took place during the daytime and that daytime sleep is not habitual for our patients, we do not know the extent to which this circadian phase of usually low sleep propensity could have contributed to our findings. A third limitation was the absence of a respiration signal coupling that could have affected HRV variables. However, slow-wave sleep has been hypothesized to be an optimal recording condition to measure HRV because of stable breathing patterns (14).

Moreover, as stated earlier, the LF/HF ratio has been challenged as a marker of the sympathovagal balance (21, 23, 49), and thus, further studies using sympathetic and parasympathetic blockade might be needed to confirm our findings. Here, we have interpreted the direction of our HRV findings in sleepwalkers' recovery slow-wave sleep during the first cycle as a marker of parasympathetic predominance. However, given the complex physiological factors affecting the LF band, our findings could be interpreted in another direction: The LF band has been suggested to be more closely related to the neural modulation rather than effect on cardiac function (21), and thus, might be of central origin, which is highly relevant in the study of sleepwalking pathophysiology. In fact, lower nLF and LF/HF in sleepwalkers' recovery slow-wave sleep could represent a reduced baroreflex sensitivity and control over cardiac function (21), which is normally the greatest during slow-wave sleep (11). This interpretation also supports an abnormal autonomic modulation of slow-wave sleep in sleepwalkers, especially during their first cycle of recovery sleep.

## Conclusions

In summary, our findings suggest that parasympathetic predominance during sleepwalkers' post sleep deprivation recovery slow-wave sleep may highlight modulation abnormalities that contribute to sleepwalkers' difficulty transitioning normally between sleep and wakefulness. The dissociation between parasympathetic dominance and reduced slow-wave sleep depth might represent the concomitant presence of wake and sleep-related characteristics. Moreover, increased sleep pressure may play an important role in the abnormal autonomic modulation observed in our sample of adult sleepwalkers. Future studies of autonomic activation during slow-wave sleep, particularly prior to somnambulistic episodes, would help clarify the role that parasympathetic activation may play in sleepwalkers' atypical arousal responses and the extent to which this activation may be implicated in the broader mechanisms believed to underlie sleepwalking pathophysiology.

## Data Availability Statement

The raw data supporting the conclusions of this article will be made available by the authors, without undue reservation.

## Ethics Statement

The studies involving human participants were reviewed and approved by Ethics Committee of the Hôpital du Sacré-Coeur de Montréal. The patients/participants provided their written informed consent to participate in this study.

## Author Contributions

GS and A-AB contributed to the design of the study, performed data processing, statistical analyses, wrote the first draft of the manuscript and integrated co-authors' comments into the submitted version. AZ and JM obtained funding for the study, conceptualized its overall design and method of data acquisition, and helped draft the original and subsequent versions of the manuscript. JC and AD contributed to the study's conception and design. All authors contributed to the revision of the manuscript, and read and approved the submitted version.

## Conflict of Interest

JM reports personal fees from Takeda, Novartis and Merck Pharmaceuticals outside the submitted work. JC reports grants from Canopy Growth, Rana, Respironics/Philips and consulting fees from Eisai, all of which are unrelated to the present work. AD received research grants from Jazz Pharma, Flamel Ireland, Biron, Canopy Growth, served on advisory boards from UCB, Eisai and Jazz Pharma and received honorarium from speaking engagement from UCB, Eisai and Paladin Labs. None of the financial disclosures are relevant to the submitted work. The remaining authors declare that the research was conducted in the absence of any commercial or financial relationships that could be construed as a potential conflict of interest.
